# Alzheimer’s Disease Projection From Normal to Mild Dementia Reflected in Functional Network Connectivity: A Longitudinal Study

**DOI:** 10.3389/fncir.2020.593263

**Published:** 2021-01-21

**Authors:** Mohammad S. E. Sendi, Elaheh Zendehrouh, Robyn L. Miller, Zening Fu, Yuhui Du, Jingyu Liu, Elizabeth C. Mormino, David H. Salat, Vince D. Calhoun

**Affiliations:** ^1^Wallace H. Coulter Department of Biomedical Engineering at Georgia Institute of Technology and Emory University, Atlanta, GA, United States; ^2^School of Electrical and Computer Engineering, Georgia Institute of Technology, Atlanta, GA, United States; ^3^Tri-Institutional Center for Translational Research in Neuroimaging and Data Science, Georgia State University, Georgia Institute of Technology, Emory University, Atlanta, GA, United States; ^4^Department of Computer Science, Georgia State University, Atlanta, GA, United States; ^5^School of Computer and Information Technology, Shanxi University, Taiyuan, China; ^6^School of Medicine, Stanford University, Palo Alto, CA, United States; ^7^Department of Neurology and Neurological Sciences, School of Medicine, Stanford University, Stanford, CA, United States; ^8^Harvard Medical School, Cambridge, MA, United States; ^9^Massachusetts General Hospital, Boston, MA, United States

**Keywords:** Alzheimer’s disease, resting state fMR imaging, hidden Markov model, longitudinal study, dynamic functional network connectivity

## Abstract

**Background:**

Alzheimer’s disease (AD) is the most common age-related problem and progresses in different stages, including mild cognitive impairment (early stage), mild dementia (middle-stage), and severe dementia (late-stage). Recent studies showed changes in functional network connectivity obtained from resting-state functional magnetic resonance imaging (rs-fMRI) during the transition from healthy aging to AD. By assuming that the brain interaction is static during the scanning time, most prior studies are focused on static functional or functional network connectivity (sFNC). Dynamic functional network connectivity (dFNC) explores temporal patterns of functional connectivity and provides additional information to its static counterpart.

**Method:**

We used longitudinal rs-fMRI from 1385 scans (from 910 subjects) at different stages of AD (from normal to very mild AD or vmAD). We used group-independent component analysis (group-ICA) and extracted 53 maximally independent components (ICs) for the whole brain. Next, we used a sliding-window approach to estimate dFNC from the extracted 53 ICs, then group them into 3 different brain states using a clustering method. Then, we estimated a hidden Markov model (HMM) and the occupancy rate (OCR) for each subject. Finally, we investigated the link between the clinical rate of each subject with state-specific FNC, OCR, and HMM.

**Results:**

All states showed significant disruption during progression normal brain to vmAD one. Specifically, we found that subcortical network, auditory network, visual network, sensorimotor network, and cerebellar network connectivity decrease in vmAD compared with those of a healthy brain. We also found reorganized patterns (i.e., both increases and decreases) in the cognitive control network and default mode network connectivity by progression from normal to mild dementia. Similarly, we found a reorganized pattern of between-network connectivity when the brain transits from normal to mild dementia. However, the connectivity between visual and sensorimotor network connectivity decreases in vmAD compared with that of a healthy brain. Finally, we found a normal brain spends more time in a state with higher connectivity between visual and sensorimotor networks.

**Conclusion:**

Our results showed the temporal and spatial pattern of whole-brain FNC differentiates AD form healthy control and suggested substantial disruptions across multiple dynamic states. In more detail, our results suggested that the sensory network is affected more than other brain network, and default mode network is one of the last brain networks get affected by AD In addition, abnormal patterns of whole-brain dFNC were identified in the early stage of AD, and some abnormalities were correlated with the clinical score.

## Introduction

Alzheimer’s disease (AD) is the most common age-related dementia, typically affecting individuals over 65 years of age (Masters et al., 2015). AD usually progresses slowly in several stages, including mild (early stage), moderate (middle stage), and severe (late stage) (Ryan and Rossor, 2011). To date, there is no way to cure AD, but some medications can decelerate its progress (Yiannopoulou and Papageorgiou, 2020). Therefore, predicting the progression from a normal stage to mild cognitive impairment and further to AD itself is an important step toward early medical intervention.

Resting-state functional magnetic resonance imaging (rs-fMRI) that indirectly measures neural processing in the brain based on the blood oxygenation can be used to identify spatially distributed networks in the brain. In recent years, functional connectivity or its network analog functional network connectivity (FNC), including dynamic (dFC/dFNC) and static (sFC/sFNC), achieved from rs-fMRI time series has uncovered a great deal of knowledge about the brain dysconnectivity in various neurological disorder including schizophrenia (Abrol et al., 2017; Sendi et al., 2020), major depression disorder (Zhi et al., 2018), autism (Cerliani et al., 2015; de Lacy et al., 2017; Mash et al., 2019), ADHD (Wang et al., 2018), and AD (Brier et al., 2014). In particular for AD, previous studies reported a reduction in the default-mode network FC in AD compared with mild cognitive impairment (MCI) patients and healthy subjects (Soman et al., 2020). Another study reported a difference in the FC of sensorimotor network (SMN), visual network (VSN), and default mode network of healthy control (HC) subjects and AD patients (Zheng et al., 2017).

By assuming that FNC is invariant, or static over time, many of the AD-related studies mentioned above have focused on sFC/sFNC and ignored dFC/dFNC. Indeed, unlike conventional static functional network connectivity (sFNC), which is obtained from the correlation within an entire time series, dFNC refers to the connectivity between any pair of brain networks within sub-intervals of time series (Calhoun et al., 2014). In fact, dFNC research suggests that cognitive deficits and clinical symptoms associated with many neurological disorders do not only depend on the strength of the connectivity between any pair of brain regions but also on the variation of connectivity strength of those regions over time (Calhoun et al., 2014; Damaraju et al., 2014; Zhi et al., 2018; Zendehrouh et al., 2020). In recent years, a few papers studied dFNC in AD. For instance, we investigated whole-brain dFNC in AD and subcortical ischemic vascular disease (SIVD) (Fu et al., 2019). Another study explored the temporal properties of dFNC associated with dementia in Parkinson’s disease (Fiorenzato et al., 2019). However, the longitudinal dFNC changes from cognitive normal to mildly then severely cognitively impaired has not been extensively explored.

In the current study, we explored the temporal dynamics of the whole-brain FNC from 1385 rs-fMRI scans of HC and very mild AD (vmAD). We used a sliding window approach followed by the k-means clustering method to identify a set of connectivity states (Calhoun et al., 2014). Next, we calculated between-state transition probability via hidden Markov model (HMM) and the amount of the time each subject spends in a state, called occupancy rate or OCR, to model the temporal properties of dFNC. We investigated the correlation between HMM and OCR features with the clinical dementia rating scale sum of boxes (CDR-SOB) scores. In addition, we explored the link between state-specific connectivity features with CDR-SOB. Finally, we trained a support vector machine (SVM) to predict from HC to vmAD based on the sFNC connectivity features and dFNC features, including HMM and OCR.

## Materials and Methods

### Participants

In this study, the data we used are from the longitudinal Open Access Series of Imaging Studies (OASIS)-3 cohort, which was collected from several ongoing studies in the Washington University Knight Alzheimer Disease Research Center over 15 years (LaMontagne et al., 2019). This data contains 1385 rs-fMRI imaging and related clinical and demographic data at the time of scanning (from 910 subjects) with age ranging from 42 to 95 years. For each subject, the imaging data, demographic, and clinical dementia rating (CDR) scale were used in any stage of cognitive functionality. All participant must have CDR ≤ 1 at the time of the clinical core assessment and once the participant reached CDR = 2 or CDR-SOB > 9, they were no longer eligible for the study (LaMontagne et al., 2019). We evaluated the cognitive stage of the participants at the time of the scanning based on the CDR-SOB scores and organized them in 2 groups, including healthy control or HC (CDR-SOB = 0), very mild AD or vmAD (0.5 ≤ CDR-SOB ≤ 9) (O’Bryant et al., 2008). In total, we have 1028 scan of HC, 357 scans of vmAD patients. The demographic information is provided in Table 1.

**TABLE 1:** Demographic and clinical information.

**HC**	*N*	1028
	Gender (M/F)	415/613
	Age	69.83 ± 8.64
	CDR-SOB	0 ± 0

**vmAD**	*N*	357
	Gender (M/F)	215/142
	Age	75.10 ± 7.85
	CDR-SOB	2.68 ± 2.10

*HC, healthy control; vmAD, very mild Alzheimer Disease; M, male; F, female; CDR-SOB, clinical dementia rating scale sum of boxes.*

### Imaging Data Acquisition

Two Trio 3T with a 20-channel head coil was used to collect imaging data (Siemens Medical Solutions United States, Inc). High resolution T2^∗^-weighted functional images were obtained by an echoplanar imaging or EP sequence with TE = 27 ms, TR = 2.2 s, flip angle = 90°;, slice thickness = 4 mm, slice gap = 4 mm, matrix size = 64, and voxel size of 1 mm × 1 mm × 1.25 mm. The duration of the scanning was 6 min.

### Data Preprocessing

**FIGURE 1:**
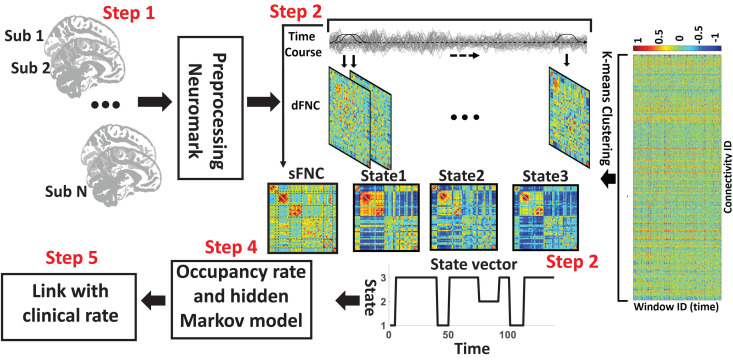
Analytic pipeline. Step1: The time-course signal of 53 ICNs have been identified using group-ICA in the Neuromak template. Step2: After identifying 53 ICNs, a taper sliding window was used to segment the time-course signals and then calculated the functional network connectivity (FNC). Each subject has 139 FNCs with a size of 53 × 53. Also, we calculated static FNC for the entire time of recording. Step3: After vectorizing the FNC matrixes, we have concatenated them, and then a k-means clustering with correlation as distance metrics was used to group FNCs to three distinct clusters. Step4: Then, based on the state vector, we calculated between-state transition probability or hidden Markov model (HMM) features and occupancy rate (OCR) for each subject. In total, nine HMM features and three OCR were estimated from the state vector of each subject. Step5: To find a link between FNC features, including sFNC and dFNC feature with clinical dementia rating scale sum of boxes (CDR-SOB), we used partial correlation by accounting for age, gender.

The analytic pipeline used in this study is shown in Figure 1. The following subsections describe the detail of this pipeline. In the first step (Step 1 in Figure 1), the first five dummy scans were discarded before preprocessing. We used statistical parametric mapping (SPM12^1^) default slice timing routines to correct differences in image acquisition time between slices. The reference slice was chosen as the slice acquired in the middle of the sequence. Rigid body motion correction was then applied to account for subject head movement, with 3-dimensional brain translations and 3-dimensional rotations estimated. Next, the imaging data underwent spatial normalization to the standard Montreal Neurological Institute (MNI) space using the echo-planar imaging (EPI) template and the default bounding box provided by the SPM toolbox and was resampled to 3 × 3× 3 mm^3^. Finally, a Gaussian kernel with a full width at half maximum (FWHM) of 6 mm was used to smooth the fMRI images.

**FIGURE 2:**
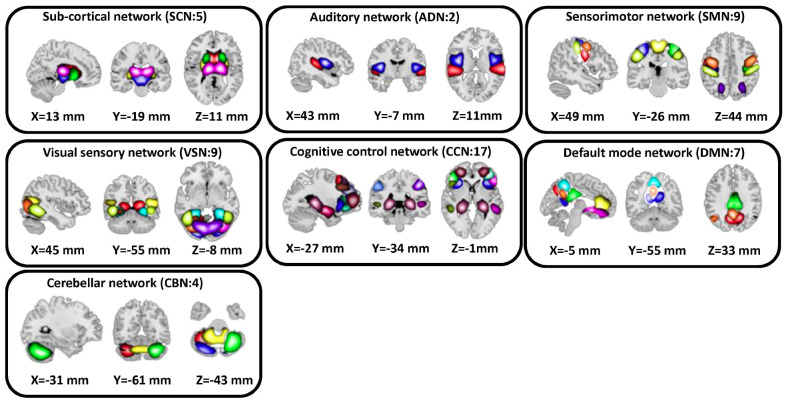
In this study, we adopted the NeuroMark pipeline to extract reliable intrinsic connectivity networks (ICNs, in total, 53 components) that are replicated across independent datasets. All 53 independent components identified by group-ICA in the Neuromark template. We put them in seven networks, including subcortical network (SCN), auditory network (AND), visual sensory network (VSN), sensorimotor network (SMN), cognitive control network (CCN), default mode network (DMN), and cerebellar network (CBN).

In this study, a set of robust network priors were used to extracted comparable components across subjects from the OASIS dataset. The network priors were extracted via the NeuroMark pipeline (Du et al., 2020; Fu et al., 2020, 2021). This framework performed group ICA with model order as 100 on two healthy controls datasets, human connectome project (HCP^2^, 823 subjects after the subject selection) and genomics superstruct project (GSP^3^, 1005 subjects after the subject selection) for creating the network priors. The extracted independent components (ICs) from the two datasets were matched by comparing the corresponding group-level spatial maps. If they show a higher spatial correlation than a given threshold (=0.4), we consider that the IC pairs were reproducible. The reproducible ICs pairs were further evaluated by examining their spatial activations and low-frequency fluctuations of their corresponding time-courses (TCs). 53 pairs of ICs were identified as meaningful and reproducible, arranging into 7 functional domains based on their anatomic and functional prior knowledge. These ICNs included subcortical network (SCN), auditory network (ADN), sensorimotor network (SMN), visual network (VSN), cognitive control network (CCN), default-mode network (DMN), and cerebellar network (CBN). The less noisy ICNs captured from the GSP dataset (Note that there were 53 ICNs from HCP which had similar spatial patterns) were chosen as the spatial network priors to back-reconstruct spatial maps and TCs for each subject. Also, to remove the remaining noise and artifact, the following post-processing procedures were performed on the time-courses signal before calculating the dynamic functional network connectivity (dFNC) between time-courses of ICs: (1) detrending linear, quadratic, and cubic trends; (2) conducting multiple regressions of the 6 realignment parameters and their temporal derivatives; (3) despiking detected outliers; and (4) low-pass filtering with a cut-off frequency of 0.15 Hz. Figure 2 shows these seven domains. Also, Table 2 shows all 53 ICNs extracted by the NeuroMark pipeline in this study.

**TABLE 2:** Component labels.

		Component name	Peak coordinate (mm)		
1	SCN	Caudate (69)	6.5	10.5	5.5
2		Subthalamus/hypothalamus (53)	–2.5	–13.5	–1.5
3		Putamen (98)	–26.5	1.5	–0.5
4		Caudate (99)	21.5	10.5	–3.5
5		Thalamus (45)	–12.5	–18.5	11.5
6	ADN	Superior temporal gyrus ([STG], 21)	62.5	–22.5	7.5
7		Middle temporal gyrus ([MTG], 56)	–42.5	–6.5	10.5
8	SMN	Postcentral gyrus ([PoCG], 3)	56.5	–4.5	28.5
9		Left postcentral gyrus ([L PoCG], 9)	–38.5	–22.5	56.5
10		Paracentral lobule ([ParaCL], 2)	0.5	–22.5	65.5
11		Right postcentral gyrus ([R PoCG], 11)	38.5	–19.5	55.5
12		Superior parietal lobule ([SPL], 27)	–18.5	–43.5	65.5
13		Paracentral lobule ([ParaCL], 54)	–18.5	–9.5	56.5
14		Precentral gyrus ([PreCG], 66)	–42.5	–7.5	46.5
15		Superior parietal lobule ([SPL], 80)	20.5	–63.5	58.5
16	VSN	Postcentral gyrus ([PoCG], 72)	–47.5	–27.5	43.5
17		Calcarine gyrus ([CalcarineG], 16)	–12.5	–66.5	8.5
18		Middle occipital gyrus ([MOG], 5)	–23.5	–93.5	–0.5
19		Middle temporal gyrus ([MTG], 62)	48.5	–60.5	10.5
20		Cuneus (15)	15.5	–91.5	22.5
21		Right middle occipital gyrus ([R MOG], 12)	38.5	–73.5	6.5
22		Fusiform gyrus (93)	29.5	–42.5	–12.5
23		Inferior occipital gyrus ([IOG], 20)	–36.5	–76.5	–4.5
24		Lingual gyrus ([LingualG], 8)	–8.5	–81.5	–4.5
25		Middle temporal gyrus ([MTG], 77)	–44.5	–57.5	–7.5
26	CCN	Inferior parietal lobule ([IPL], 68)	45.5	–61.5	43.5
27		Insula (33)	–30.5	22.5	–3.5
28		Superior medial frontal gyrus ([SMFG], 43)	–0.5	50.5	29.5
29		Inferior frontal gyrus ([IFG], 70)	–48.5	34.5	–0.5
30		Right inferior frontal gyrus ([R IFG], 61)	53.5	22.5	13.5
31		Middle frontal gyrus ([MiFG], 55)	–41.5	19.5	26.5
32		Inferior parietal lobule ([IPL], 63)	–53.5	–49.5	43.5
33		Left inferior parietal lobue ([R IPL], 79)	44.5	–34.5	46.5
34		Supplementary motor area ([SMA], 84)	–6.5	13.5	64.5
35		Superior frontal gyrus ([SFG], 96)	–24.5	26.5	49.5
36		Middle frontal gyrus ([MiFG], 88)	30.5	41.5	28.5
37		Hippocampus ([HiPP], 48)	23.5	–9.5	–16.5
38		Left inferior parietal lobue ([L IPL], 81)	45.5	–61.5	43.5
39		Middle cingulate cortex ([MCC], 37)	–15.5	20.5	37.5
40		Inferior frontal gyrus ([IFG], 67)	39.5	44.5	–0.5
41		Middle frontal gyrus ([MiFG], 38)	–26.5	47.5	5.5
42		Hippocampus ([HiPP], 83)	–24.5	–36.5	1.5
43	DMN	Precuneus (32)	–8.5	–66.5	35.5
44		Precuneus (40)	–12.5	–54.5	14.5
45		Anterior cingulate cortex ([ACC], 23)	–2.5	35.5	2.5
46		Posterior cingulate cortex ([PCC], 71)	–5.5	–28.5	26.5
47		Anterior cingulate cortex ([ACC], 17)	–9.5	46.5	–10.5
48		Precuneus (51)	–0.5	–48.5	49.5
49		Posterior cingulate cortex ([PCC], 94)	–2.5	54.5	31.5
50	CBN	Cerebellum ([CB], 13)	–30.5	–54.5	–42.5
51		Cerebellum ([CB], 18)	–32.5	–79.5	–37.5
52		Cerebellum ([CB], 4)	20.5	–48.5	–40.5
53		Cerebellum ([CB], 7)	30.5	–63.5	–40.5

### Functional Network Connectivity

The sFNC of each subject was calculated by computing the Pearson correlation between any pair of ICNs time series. With 53 ICNs, it resulted in 1378 whole-brain correlation values for each subject. In addition, for each subject i = 1 … N, the dynamic FNC (dFNC) of the whole brain was estimated via a sliding window approach, as shown in Figure 1. A tapered window obtained by convolving a rectangle (window size = 20 TRs = 44 s) with a Gaussian (σ = 3) was used to localize the dataset at each time point. It is worth mentioning that previous studies suggested that a window size between 30 and 60 s is a reasonable choice for capturing the dFNC fluctuation (Preti et al., 2017). Based on this past work, we used the 44 s as the window size. A covariance matrix, based on Pearson correlation, was calculated to measure the dFNC between ICs. The dFNC estimates of each window for each subject were concatenated to form a (C × C × T) array (where C = 53 denotes the number of ICNs and *T* = 139), which represented the changes in brain connectivity between ICNs as a function of time (Step 2 in Figure 1; Calhoun et al., 2014).

### Clustering and dFNC Latent Features

We used k-means clustering to partition dFNC window into a set of separated clusters (states). Based on the elbow criterion (the ratio of within to between cluster distance), we found that the optimal number of clusters (i.e., k) is 3. We used correlation based on Pearson correlation as a distance metric in the clustering algorithm in 1000 iterations (Allen et al., 2014; Calhoun et al., 2014) (Step 3 in Figure 1). The output of this step is 3 states for all subjects and subject-specific state vector. The state vector shows that the state of the whole-brain FNC of each subject at a specific time. In the next step, we calculated the between-state transition probability based on HMM. The transition probability, a_*ij*_, is the probability of the system to transition from state j at time t to state i at time t+1.

(1) $$a_{\mathrm{ij}}=p\left(s\left(t+1\right)=i|s\left(t\right)=j\right)$$

In addition, we computed the OCR of dFNCs in each state (Step 4 in Figure 1). In addition, for each subject, we averaged all dFNC belongs to a state as her/his state-specific FNC. In more detail, each subject has multiple dFNC in each state. Then, in each state, we used the average of dFNC (i.e., the average of 1378 connectivity features) of each subject as her/his state-specific FNC.

### Statistical Analysis

To assess the link between dFNC features, including state-specific FNC, OCR, and HMM with CDR-SOB, we used partial correlation by accounting for age and gender. We performed statistical analysis on all 1378 whole-brain connectivity features, 9 HMM features, and 3 OCR features, separately (Step 5 in Figure 1). All *p* values have been adjusted by the Benjamini-Hochberg correction method for multiple comparisons (Benjamini and Hochberg, 1995). The number of null hypothesis in state-specific FNC, OCR, and HMM were 1378, 3, and 9, respectively.

### Dementia Progression Is Associated With Functional Network Connectivity

In the next step, we explored whether functional network connectivity, including sFNC and dFNC features, can predict the progression of AD. We put subjects into two different groups. The first group contains those subjects who remained in HC, whom we call them unconverted HC or uc-HC stage within the next 5 years of the first scan, and the second group contains those subjects which their cognitive functionality changed from HC to vmAD (0.5 ≤ CDR-SOB ≤ 9), and we call c-HC. The first group contains 85 subjects (48 females and 37 males) with the mean age at 74.6478 and range between 65 and 85. The second group contains 40 subjects (18 females and 22 males) in which the mean age is 74.6878, and the range of the age is between 64 and 85. We did not observe a significant difference between the age and gender of these two groups (age: Cohen’s d = 0.16, two-sample *t* test *t*(123) = −0.90, *p* = 0.36; gender: Cohen’s d = 0.22, two-sample *t* test *t*(123) = 1.19, *p* = 0.23). We trained a SVM based on sFNC, OCR, and HMM features from the baseline rs-fMRI (around 5 years prior to the conversion) to differentiate these two groups.

One major problem in this classification is imbalanced datasets. To deal with this problem, we have used a data augmentation method called adaptive synthetic (ADASYN) sampling approach. In this method, we adaptively generated synthetic data for the minatory class based on the distribution of both classes. ADASYN generates synthetic data for the part of minority class that is harder to learn than those minority samples, which are easier to learn. In this study, we have a dataset with 85 samples in major class and 40 samples in minor class. Using ADASYN, we generated 45 samples of synthetic data for the minor class to make the dataset balanced (He et al., 2008). We trained an SVM with polynomial kernel function, as shown in Eq. 2. to classify two classes (Cortes and Vapnic, 1995).

(2) $$k\left(x_{1},x_{2}\right)={\left(1+x_{1}^{{}^{\prime }}x_{2}\right)}^{p}$$

where *p* is a positive integer value.

There are a few advantages of using SVM. (1) SVM works well for high dimensional data. (2) SVM is effective when the number of the sample is smaller than the number of dimensions, which is a common problem for neuroimaging data. (3) SVM can handle nonlinearity in the data using a kernel trick. This provides an advantage over linear classifiers like logistic regression. However, choosing the appropriate kernel function is not easy. In addition, due to the limited number of samples, it is not advisable to use a neural network classifier. It is worth mentioning that an imbalanced dataset is a challenging problem in SVM classification (Cervantes et al., 2020). Our study used ADASYN to generate synthetic data of the minatory class to elevate this problem. In more detail, we used the ADASYN-based sample and a subset of the major class (i.e., uc-HC, *N* = 45, the ADASYN sample size) and trained a model and then we tested that model on real unseen data from both uc-HC and c-HC groups. We iterate this 10 times. This number was chosen arbitrary. It worth mentioning that changing the number of iterations would not change the classification result. In each iteration, we used five-fold cross-validation method in which we used 80% of the training data to train a model and 20% of the data to validate that. The hyperparameters of the SVM classifier were selected trough an optimization process.

We calculated the classification accuracy, sensitivity, specificity, and area under the receiver operating characteristic curve (AUC) to assess the classification performance. Accuracy, sensitivity, and specificity were quantified by:

(3) $$\mathrm{Accurcy}=\frac{\mathrm{TP}+\mathrm{TN}}{\mathrm{TP}+\mathrm{FN}+\mathrm{TN}+\mathrm{FP}}$$

(4) $$\mathrm{Sensitivity}=\frac{\mathrm{TP}}{\mathrm{TP}+\mathrm{FN}}$$

(5) $$\mathrm{Specificity}=\frac{\mathrm{TN}}{\mathrm{TN}+\mathrm{FP}}$$

where TP, FN, TN, and FP denoted the number of uc-HC subjects correctly predicted, the number of c-HC subjects classified as uc-HC subject, the number of uc-HC correctly predicted, and the number of un-HC subjects classified as c-HC subject, respectively.

## Results

### Dynamic Functional Connectivity States

**FIGURE 3:**
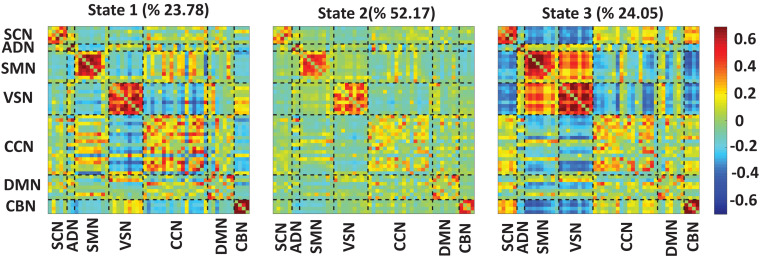
Dynamic functional connectivity states results. The three identified dFNC states using the k-means clustering method. We found strong connectivity within-ADN, within-SMN, and within-VSN in all states. We found strong connectivity between SMN and VSN in state3. Also, this state showed negative connectivity between sensory networks, including ADN, SMN, and VSN, with the rest of the brain. We found all subjects spend 23.78, 52.17, and 24.05% in state 1, state 2, and state 3, respectively. The color bar shows the strength of the connectivity. SCN, Subcortical network; ADN, auditory network; SMN, sensorimotor network; VSN, visual network; CCN, cognitive control network; DMN, default-mode network; and CBN, cerebellar network.

Figure 3 shows the reoccurring connectivity states identified by the k-means clustering method. In all states, we observed strong positive connectivity within SMN and VSN, ad CBN. State 3 showed the strongest connectivity within SMN and within VSN among all states. In addition, this state had the highest connectivity between SMN and VSN. Also, this state was separated from other states by showing the lowest negative connectivity between SMN and VSN with the rest of the brain. State 1 showed the lowest connectivity between SMN and VSN. Finally, we measured the OCR of each subject in state1, state2, and state 3. OCR represents the amount of time each subject spends in each state. Results showed that subjects spent an average of 23.78, 52.17, and 24.05% in state 1, state 2, and state 3, respectively.

### The Correlation Between State-Specific FNC and CDR-SOB

**FIGURE 4:**
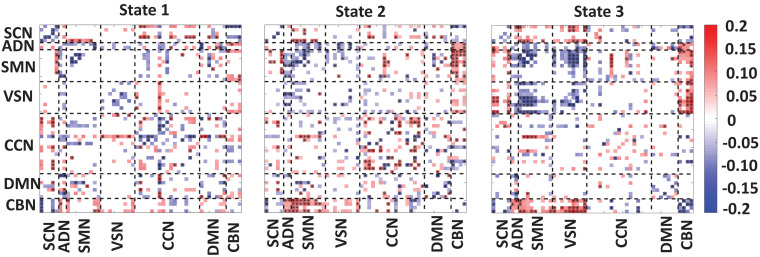
Correlation between FNC of each state and clinical score. A significant negative correlation between within-SCN, within-SMN, within-VSN, and within-CBN connectivity and the clinical dementia rating scale sum of boxes (CDR-SOB) scores. A disrupted pattern (i.e., both positive and negative) correlation was found between within-CCN and within DMN with CDR-SOB. A similar disrupted pattern was observed between CDR-SOB and between-networks connectivity. However, we consistently observed negative connectivity between CDR-SOB and the connectivity among sensory networks. The color bar shows the strength of the connectivity. SCN, subcortical network; ADN, auditory network; SMN, sensorimotor network; VSN, visual network; CCN, cognitive control network; DMN, default-mode network; and CBN, cerebellar network. The significant correlation that passes the multiple comparisons is marked by asterisks.

Figure 4 showed the partial correlation between state-specific FNC and CDR-SOB while we controlled for age and gender. The significant correlations (uncorrected *p* < 0.05) are shown in red (positive correlation) and blue (negative correlation). Also, a significant correlation that passes the multiple comparisons is marked by asterisks.

In state 1, we observed a significant and negative correlation between within-SCN, within-SMN, within-VSN connectivity, and CDR-SOB. This means that this connectivity decreases by progression from normal to mild dementia states. In this state, we found a reorganized (i.e., both positive and negative) patterns in the correlation between within-DMN connectivity and within CCN-connectivity with CDR-SOB. A similar reorganized correlation pattern was observed between the between-network connection and CDR-SOB. Specifically, a more reorganized pattern was observed in the correlation between SCN connectivity with the rest of the brain.

In state 2, similar to state 1, we observed a negative correlation between the connectivity of within-SCN, within-SMN, within-VSN, within-CBN connectivity with CDR-SOB. We also observed both positive and negative correlations between the connectivity of CCN and DMN and CDR-SOB. Compared with the other states, this state showed a more significant correlation of within-CCN connectivity and CDR-SOB, in which many of them were positive. Also, within-DMN connectivity showed more negative connectivity compared with that of other states. This state also showed a positive correlation between connectivity between SMN and CBN and CDR-SOB. Also, we observed both positive and negative correlations between CDR-SOB and between-network connectivity in state 2.

State 3 showed a significant and negative correlation between the within-SMN, within-VSN, within-DMN, within-CCN, and within-CBN connectivity and CDR-SOB. The amount of significant correlation between within-SMN, within-VSN, and between SMN and VSN connectivity with CDR-SOB was more than those of the other states. Also, this state showed a significant positive correlation between VSN and CBN connection and CDR-SOB. Overall, we observed a reorganized pattern in the correlation between CDR-SOB and between-network connection in this state.

### The Correlation Between Temporal Properties of dFNC and CDR-SOB

We calculated the partial correlation between CDR-SOB and temporal features of dFNC (i.e., OCR and HMM) by controlling the age and gender. We found a positive correlation between OCR of state 1 and CDR-SOB (*r* = 0.07, corrected *p* = 0.009) and a negative correlation between OCR of state 3 and CDR-SOB (*r* = −0.14, corrected *p* = 2e^–7^). Also, we observed a negative correlation between CDR-SOB and a_11_, i.e., the transition from state 1 to state 1 (*r* = 0.07, corrected *p* = 0.02), and a positive correlation between CDR-SOB and a_33_, i.e., the transition within state 3 (*r* = −0.11, corrected *p* = 0.0001).

### Both Healthy and Patient Brain Follow Similar State Pattern

**FIGURE 5:**
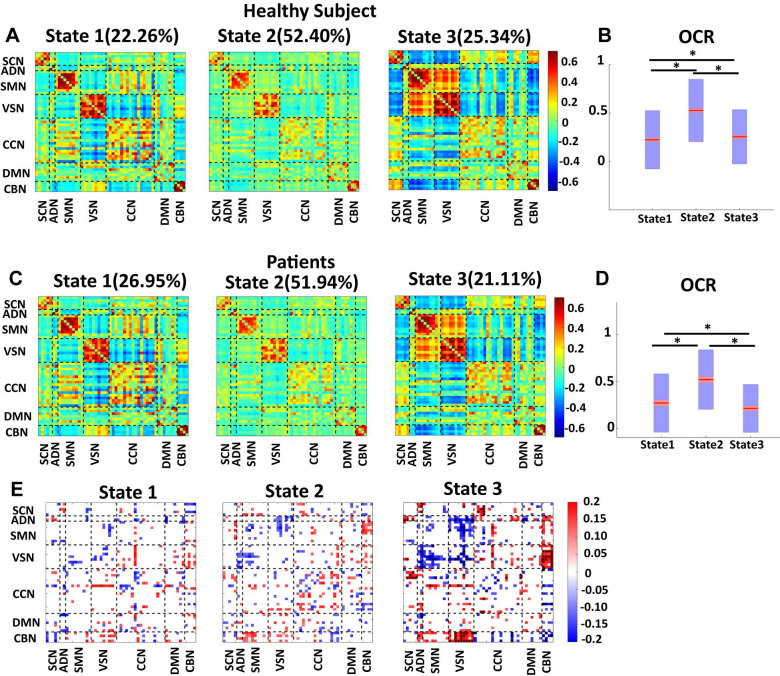
Dynamic functional network connectivity in healthy control and patients. **(A)** The three identified dFNC states using the k-means clustering method in healthy control (HC). **(B)** Occupancy rate (OCR) of HC in each state. HCs have the highest OCR in state 2 (corrected *p* < 0.001). OCR of state 3 is higher than the OCR of state 1 in HC subjects (corrected *p* < 0.01). **(C)** The three identified dFNC states using the k-means clustering method in patients (vmAD). **(D)** Occupancy rate (OCR) of HC in each state. vmAD subjects have the highest OCR in state 2 (corrected *p* < 0.001). OCR of state 1 is higher than OCR of state 2 in patients (corrected *p* < 0.01). **(E)** Correlation between FNC of each state and clinical score in the only patient group. SCN, subcortical network; ADN, auditory network; SMN, sensorimotor network; VSN, visual network; CCN, cognitive control network; DMN, default-mode network; and CBN, cerebellar network. The significant correlation that passes the multiple comparisons is marked by asterisks.

Since the number of HC scan is more than the number of patient ones, we applied the clustering method to their dFNC of HC and patients, separately. The results are shown in Figure 5. This figure shows that our approach captured a similar brain state in both groups. We used the Pearson correlation between states’ FNC to assess the similarity between them. The correlation between state 1 of HC with state 1 of the patient group, between state 2 of HC with state 2 of the patient group, and between state 3 of HC with state 3 of the patient group were 0.9903 (*N* = 1378, *p* < 0.001), 0.9825 (*N* = 1378, *p* < 0.001), and 0.9921 (*N* = 1378, *p* < 0.001), respectively (Figures 5A,B). In addition, the OCR followed a similar pattern with the results when we concatenated all subjects. State2 shows the highest OCR among all three states in both groups. HC subjects have higher OCR in state 1 than patients (*p* < 0.01), while patients have higher OCR in state 3 than that of HC subjects (*p* < 0.01) (Figures 5C,D). These results proved that the HC subject’s dFNC did not dominate the state pattern. In addition, to reference the states easier in this paper, we put a name on each state. Since both healthy subjects and patients spend more than 50% of their scanning time in state 2, we called this state a baseline. Since vmAD patients spend more time in state1, we called this state the vmAD-related state, and finally, we call state 3, which healthy subjects spend more than in this state than state 2, as an HC-related state. Only in state 3, we observed a significant link between CDR-SOB and FNC after FDR correction (shown by asterisks). We observed a decrease in sensory network FNC by AD’s progression. Also, we observed a reorganized pattern in the CCN FNC pattern.

### Changing the Number of States Does Not Change the Results

**FIGURE 6:**
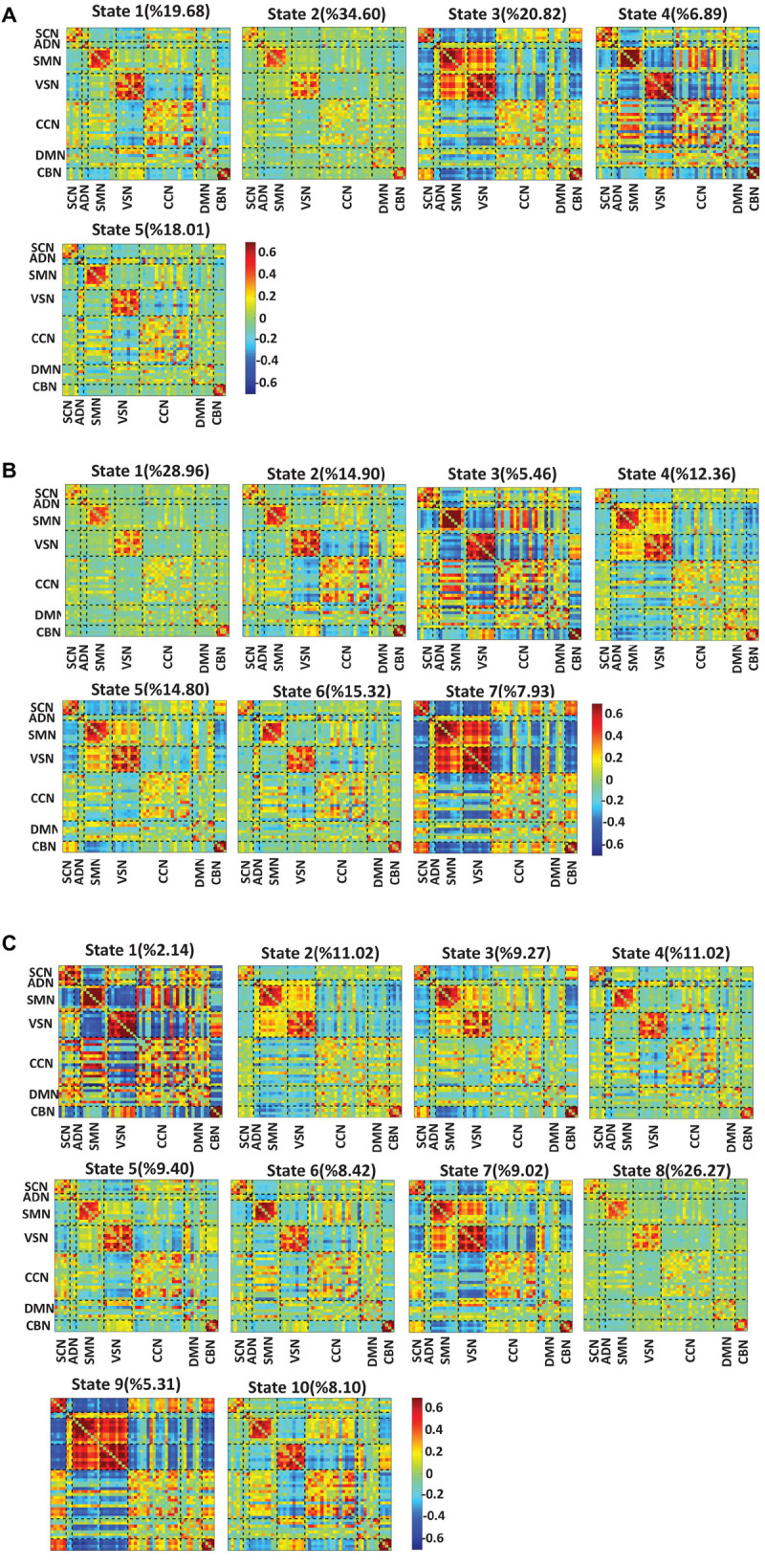
Dynamic functional connectivity states result with different *k* values. **(A)** The dFNC states identified using the k-means clustering method with *k* = 5. **(B)** The dFNC states identified using the k-means clustering method with *k* = 7. **(C)** The dFNC states identified using the k-means clustering method with *k* = 10. The color bar shows the strength of the connectivity. SCN, subcortical network; ADN, auditory network; SMN, sensorimotor network; VSN, visual network; CCN, cognitive control network; DMN, default-mode network; and CBN, cerebellar network.

To test whether the number of clusters (or states) would change the results or not, we applied the same clustering method for *k* = 5, *k* = 7, and *k* = 10. Figures 6A–C show the results for *k* = 5, *k* = 7, and *k* = 10, respectively. We observed similar state patterns with different *k* values. State 1 (*k* = 3) is similar to state 1(*k* = 5), state 2(*k* = 7) and state 5(*k* = 10). State 2 (*k* = 3) is similar to state 2(*k* = 5), state 1 (*k* = 7), and state 8 (*k* = 10). State3 (*k* = 3) is similar to state 3(*k* = 5), state 5 (*k* = 7), and state7 (*k* = 10). Increasing k above the optimized value of *k* = 3 yields states whose similarity to those in the optimized value weakens as k grows. However, the two states (states 1 and 3) in the optimized clustering whose OCR is significantly linked to CDR-SOB are highly replicable up to *k* = 7 and whose occupancy has a replicable significant relationship to CDR-SOB. Table 3 shows the correlation between CDR-SOB with OCR of clustering with different *k* values.

**TABLE 3:** The link between occupancy rate of each state and CDR-SOB.

	State1	State2	State3	State4	State5	State6	State7	State8	State9	State10
***K* = 3**	**0.07**	0.02	**−0.14**	NA	NA	NA	NA	NA	NA	NA
	**(0.013)**	(0.09)	**(6e-7)**							
***K* = 5**	**0.088**	–0.014	**−0.083**	0.033	–0.017	NA	NA	NA	NA	NA
	**(0.001)**	(0.580)	**(0.001)**	(0.026)	0.513					
***K* = 7**	−3e-4	**0.100**	–0.013	–0.036	**−0.075**	0.043	–0.053	NA	NA	NA
	(0.991)	**(0.001)**	(0.722)	(0.241)	**(0.017)**	(0.188)	(0.106)			
***K* = 10**	−7.8e-4	–0.030	**−0.073**	0.060	**0.090**	–0.009	–0.057	–0.010	–0.040	0.054
	(0.771)	(0.368)	**(0.030)**	(0.080)	**(0.008)**	(0.771)	(0.082)	(0.771)	(0.219)	(0.087)

*The bold values are the significant correlation after multiple comparisons. All corrected p values are in the parentheses.*

### Dementia Progression Associated With Functional Network Connectivity

Using baseline FNC features, including sFNC, HMM, and OCR, from whole-brain FNC, we successfully predicted the conversion from the normal state to vmAD by classifying those subjects converted to the mildly impaired stage (i.e., c-HC) from those stayed unchanged within 5 years, i.e., uc-HC. The average accuracy, sensitivity, specificity, and AUC in this classification was 75, 72, 78, and 81%, respectively.

## Discussion

In this study, we explored the dynamic of whole-brain FNC of HC (CDR-SOB = 0) and vmAD (0.5 ≤ CDR-SOB ≤ 9) subjects from the longitudinal rs-fMRI OASIS-3 (LaMontagne et al., 2019). Using a data-driven approach, we extracted 53 ICs for the whole brain and used a sliding window approach followed by a clustering method to study dFNC of this dataset. We found that the connectivity between VSN and SMN is dynamic by a transition from positive connectivity in state 3 to moderate positive connectivity in state 2 and negative connectivity in state 1. In addition, the connectivity between SMN and VSN with the rest of the brain changes from negative connectivity in state 3 to other to state with more positive connectivity. Besides SMN and VSN, we found a dynamical pattern in CCN connectivity. Overall, we found the whole-brain FNC is highly dynamic. This result argues the previous AD-related literature that mainly ignored the dynamic behavior of brain connectivity. Although a few studies explored the dFNC recently (Schumacher et al., 2019; Gu et al., 2020), the current study uses Neuromark as a replicable platform to extract data-driven ICs from relatively large longitudinal data. This replicable platform can generate ICs and replicate that across the different datasets. This would help reproduce similar brain states for FNC across different datasets, which is very important when studying dynamics (Iraji et al., 2020).

We found within-SMN FC decreased by the transition from a HC to a vmAD. This pattern was observed in all 3 states. Previous studies showed a decrease in SMN FC is shown in AD patients compared with that of HC subjects (Agosta et al., 2010; Damoiseaux et al., 2012; Wang P. et al., 2015; Li et al., 2020). In more detail, we found the FC within the postcentral gyrus decrease in vmAD than the HC brain. Postcentral gyrus has a key role in somatic sensation, including pain and temperature (DiGuiseppi and Tadi, 2019). Also, a previous study showed an impairment of pain and temperature sensation in mild dementia (Fletcher et al., 2015). Therefore, dysconnectivity in postcentral gyrus can potentially explain the impairment of somatic sensation in the early stage of dementia and suggest a prospective study.

In addition, we observed that the VSN FC, in the particular fusiform gyrus, decreases when the brain progress from the healthy state to mild dementia. As previous studies showed, the fusiform gyrus is involved in face recognition, and alteration in the connectivity between this brain region and other subregions of VSN causes impairment in face recognition (Fur et al., 2011; Cai et al., 2015). Functional dysconnectivity between fusiform and the rest of the VSN potentially can explain the impairment of face recognition in the early stage of AD progression (Uhlmann et al., 1991). Also, results indicated reduced FC among the brain sensory networks, i.e., ADN, SMN, and VSN, by progression from HC state to vmAD. Information processing integration of multisensory signals is a hallmark of self-awareness. For instance, (Ehrsson, 2007) showed that the matching between visual perception and proprioceptive signals is necessary for preserving the self-consciousness. Disconnection among sensory networks in mild dementia patients than that of healthy subjects can potentially explain the underlying mechanism of self-awareness discrepancy in AD patients. The current findings suggest future studies for exploring a causal link between dysconnectivity in the sensory network and lack of self-awareness in AD patients.

We also observed a disrupted temporal and spatial pattern in the connectivity between CBN and other brain networks. In all states, we found a decrease in the connectivity between CBN and SCN by advancement from the HC brain to vmAD. However, the connectivity between CBN and SMN, and between CBN and VSN are higher in vmAD than the HC subjects. This finding is consistent with a previous study that showed a reorganized pattern in the connectivity between cerebellar subregions and DMN, VSN, SMN (Zheng et al., 2017). However, we did not detect a significant pattern in the correlation between the clinical rate and the connectivity between CBN and DMN.

In addition, we found a disrupted pattern in DMN connectivity by having reduced connectivity in state 3, and both increased and reduced connectivity in state 1 and state 2 for vmAD than the normal brain. Based on sFC, previous studies reported both increase (Tao et al., 2017) and decrease (Binnewijzend et al., 2012) in whole-DMN connectivity of the AD subject. Another study reported no significant difference in DMN connectivity between AD patients with HC (Grieder et al., 2018). Although a small sample size might affect the statistical power, as previously shown in a study of major depression (Yan et al., 2019), this inconsistent result partially could be due to focusing on static FC, which is obtained from the correlation within an entire time series. Similarly, we observed a disrupted spatial and temporal pattern in CCN connectivity. In addition, we found a reorganized pattern in the connectivity between CCN and other networks such as SCN, ADN, SMN, VSN, DMN, and CBN. A recent study showed a reorganized pattern in the connectivity between inferior parietal lobule, as a part of CCN, with default mode, salience, executive control, and SMNs (Wang Z. et al., 2015). Our new finding provides new knowledge about the reorganized pattern between CNN and the rest of the brain. The decrease in the CCN FC might explain the loss in the functional integrity of the CCN network, and the increased FC showed that vmAD patients potentially utilize additional brain subregions to compensate for the impairment of cognitive function. We also found that the FC within SCN, including caudate, thalamus, and putamen, decreased in vmAD patients compared with that of normal subjects. This result is consistent with a previous study that showed subjects with the risk of AD showed less connectivity in caudate and thalamus (Li W. et al., 2014).

Next, we calculated the correlation between clinical rate and OCR and HMM. We found HC subjects spend more time in state 3, which showed the highest positive connectivity among sensory networks, i.e., VSN, SMN, and ADN, is less in vmAD patients than the normal subjects. Also, we found the dwell time of state1, which showed the least connectivity among sensory networks, is higher for the patients with mild dementia. This finding provides further evidence of the effect of disease on the dysregulating temporal properties of FNC. A recent study showed that AD subjects spend more time in a sparsely connected state in which the motor network isolated from the rest of the brain (Schumacher et al., 2019). Our result is consistent with the study mentioned above by showing that the subjects with mild severity in the early stage of AD spend more time in state 1, which shows sparse connectivity among brain networks. However, another part of our result that shows normal brain spend more time in state 3, in which SMN is isolated from most parts of the brain except VSN, contradicts the result of the aforementioned study. In addition, spending more time in a state with lower connectivity between SMN and VSN and less time in a state with stronger connectivity between SMN and VSN by subject in the early stage of AD emphasizes more on the role of this connectivity by the transition from the normal stage to the early stage of AD. Also, our result is consistent with another study from our group on a different dataset that showed that AD patients spend more time in the state with lower connectivity and spend less time in a state with higher connectivity (Du et al., 2019).

Prior work has demonstrated the regional patterns of AD pathology and their overlap with DMN regions (Desgranges et al., 2011). Therefore, we expected DMN to be impacted, as demonstrated in prior studies. However, we found that associations between primary sensory/motor networks were most correlative to symptom severity. Sensory and motor networks are considered relatively spared from AD pathology, at least until later stages of the disease. These exciting findings may suggest that although relatively preserved and potentially due to high signals in these regions, regions involved in cross-modal sensory/motor integration are damaged. This information provides a sensitive measure of neural damage in AD (potentially more sensitive than primary degeneration regions). Our result might suggest that DMN is the last brain network that is affected by AD. Our result might also explain the previous study’s finding that motor function changes might predate the cognitive impairments and dementia onset (Verghese et al., 2007; Wesson et al., 2011). However, a prospective study is needed to find which specific sensory or motor function changes sign early AD. Also, our new result about dysconnectivity in the somatosensory network might explain why physical exercise would prevent AD (De la Rosa et al., 2020) by increasing FNC among sensory networks (Demirakca et al., 2016).

Neurofeedback is a form of real-time biofeedback regulating brain activity and promoting brain function and behavioral performance (Omejc et al., 2019). In this technique, the neural signals are recorded from the brain. A feedback mechanism is then used to control the neural signal features through a feedback loop in the form of audio, video, or a combination of them. This closed-loop therapy has been widely used for major depressive disorder (Lee et al., 2019), attention deficit hyperactivity disorder (Enriquez-Geppert et al., 2019), and autism (Eroglu and Ekici, 2020) and got attention for treating AD in recent years. A recent study used the amount of delta, theta, alpha, and beta activity from EEG signal as a control signal in neurofeedback to improve cognitive function in AD (Luijmes et al., 2016). We introduced the sensory network’s connectivity as a potential control marker in the neurofeedback in the current study. More specifically, our result suggests a possible benefit of administering the neurofeedback during the vmAD-related state and switching the brain state from vmAD-related to HC-related state. Although many technical limitations of real-time implanting neurofeedback system integrated with dFNC exist (Monti et al., 2017; Watanabe et al., 2017). Our results suggest a future benefit of dFNC states in neurofeedback in AD treatment.

Finally, we show that both dFNC and sFNC can be used to predict the conversion from healthy to vmAD based on their baseline recording. Previous literature proposed a few models to predict conversion from MCI to AD (Risacher et al., 2009; Li H. et al., 2014; Abrol et al., 2020). For example, one study used 75 state MCI, i.e., who did not convert to AD, and 51 progressive MCI, i.e., who changed to AD within 3 years, modeled a SVM and could classify them with 79.37% accuracy based on the brain connectivity features (Zheng et al., 2019). Another study used structural and genetic data for prediction from converted normal subjects to mild cognitive impairment from the unconverted normal subject within 5 years and could predict the conversion from normal to mild cognitive impairment with AUC of 85% (Albert et al., 2018). However, the model for the conversion from the normal brain to mild impairment state based on their baseline recording has not extensively reported. The current study shows a potential for FNC in predicting from healthy aging to mild impairment stage.

### Limitations and Future Study

There are some limitations to this work. The choice of window size is an implicit assumption about the dynamic behavior in that a short window captures more rapid fluctuations, whereas a longer window does more smoothing. Future work can be accomplished to evaluate the range of dynamics more comprehensively (Faghiri et al., 2020). In addition, we used SVM to compute the classification between individuals who converted to vmAD, and those did not convert. Other more advanced methods like neural network-based classification can potentially increase the prediction accuracy. However, applying neural network-based classification is almost impossible due to the limited number of samples in the longitudinal data used in this classification.

## Conclusion

In work reported here, we extend this existing body of knowledge into the dynamic realm, investigating how time-varying properties of whole-brain FNC changes by the transition from healthy aging to vmAD. We found a state-specific reorganized pattern in the whole-brain FNC of vmAD patients. We observed a decreased connectivity among sensory networks, including SMN, VSN, and ADN, in mild dementia state. This provides a piece of new knowledge about the sensory network dysconnectivity in the early stage of AD with mild symptom severity. This potentially marked that sensory network is one of the brain networks that got affected more than the other brain network in the early stage of AD. In addition, we found a reorganized pattern, i.e., both increase and decrease in DMN and CCN connectivity. A similar changed pattern was observed in between-networks connectivity. We also found that mild dementia is linked to the temporal pattern on FNC by increasing the amount of the time staying in a sparsely connected state with lower functional connectivity among sensory networks. These results emphasized that not only the transition from the normal state to mild dementia changes the connectivity strength, but also it dysregulates the temporal properties of FNC.

## Data and Code Availability Statement

The datasets for this study from Open Access Series of Imaging Studies (OASIS)-3 (https://www.oasis-brains.org/). The preprocessed data are available from the corresponding author upon reasonable request. The code used for preprocessing and dFNC calculation are available at https://trendscenter.org/software/. For generating the synthetic data, we used the code provided by Dominic Siedhoff (2020). ADASYN (improves class balance, an extension of SMOTE) (https://www.mathworks.com/matlabcentral/fileexchange/50541-adasyn-improves-class-balance-extension-of-smote).

## Ethics Statement

The studies involving human participants were reviewed and approved by in this study, ethical approval was granted by the relevant Ethics Committees, and informed consent was obtained from each subject prior to scanning according to the Institutional Review Board of Washington University School of Medicine. The patients/participants provided their written informed consent to participate in this study.

## Author Contributions

MS developed the study, conducted data analysis, interpreted the results, and wrote the original manuscript draft. EZ conducted data analysis and wrote the manuscript draft. ZF and YD conducted data analysis and edited the original draft. JL, RM, EM, and DS edited the original draft and provided a critical review of the initial draft. VC developed the study, interpreted the results, edited the original draft, and provided critical review to the initial draft. All authors approved the final manuscript.

## Conflict of Interest

The authors declare that the research was conducted in the absence of any commercial or financial relationships that could be construed as a potential conflict of interest. The reviewers SK and AC-P declared a shared affiliation with several of the authors, MS and VC, respectively, to the handling editor at the time of review.

## Footnotes

1 https://www.fil.ion.ucl.ac.uk/spm/

2 https://www.humanconnectome.org/study/hcp-young-adult/document/1200-subjects-data-release

3 https://dataverse.harvard.edu/dataverse/GSP
